# Comprehensive Acute Kidney Injury Survivor Care: Protocol for the Randomized Acute Kidney Injury in Care Transitions Pilot Trial

**DOI:** 10.2196/48109

**Published:** 2023-05-22

**Authors:** Heather P May, Joan M Griffin, Joseph R Herges, Kianoush B Kashani, Andrea G Kattah, Kristin C Mara, Rozalina G McCoy, Andrew D Rule, Angeliki G Tinaglia, Erin F Barreto

**Affiliations:** 1 Mayo Clinic Rochester, MN United States

**Keywords:** acute kidney injury, acute renal failure, care transitions, chronic kidney disease, nephrologists, randomized controlled trials

## Abstract

**Background:**

Innovative care models are needed to address gaps in kidney care follow-up among acute kidney injury (AKI) survivors. We developed the multidisciplinary AKI in Care Transitions (ACT) program, which embeds post-AKI care in patients’ primary care clinic.

**Objective:**

The objective of this randomized pilot trial is to test the feasibility and acceptability of the ACT program and study protocol, including recruitment and retention, procedures, and outcome measures.

**Methods:**

The study will be conducted at Mayo Clinic in Rochester, Minnesota, a tertiary care center with a local primary care practice. Individuals who are included have stage 3 AKI during their hospitalization, do not require dialysis at discharge, have a local primary care provider, and are discharged to their home. Patients unable or unwilling to provide informed consent and recipients of any transplant within 100 days of enrollment are excluded. Consented patients are randomized to receive the intervention (ie, ACT program) or usual care. The ACT program intervention includes predischarge kidney health education from nurses and coordinated postdischarge laboratory monitoring (serum creatinine and urine protein assessment) and follow-up with a primary care provider and pharmacist within 14 days. The usual care group receives no specific study-related intervention, and any aspects of AKI care are at the direction of the treating team. This study will examine the feasibility of the ACT program, including recruitment, randomization and retention in a trial setting, and intervention fidelity. The feasibility and acceptability of participating in the ACT program will also be examined in qualitative interviews with patients and staff and through surveys. Qualitative interviews will be deductively and inductively coded and themes compared across data types. Observations of clinical encounters will be examined for discussion and care plans related to kidney health. Descriptive analyses will summarize quantitative measures of the feasibility and acceptability of ACT. Participants’ knowledge about kidney health, quality of life, and process outcomes (eg, type and timing of laboratory assessments) will be described for both groups. Clinical outcomes (eg, unplanned rehospitalization) up to 12 months will be compared with Cox proportional hazards models.

**Results:**

This study received funding from the Agency for Health Care Research and Quality on April 21, 2021, and was approved by the Institutional Review Board on December 14, 2021. As of March 14, 2023, seventeen participants each have been enrolled in the intervention and usual care groups.

**Conclusions:**

Feasible and generalizable AKI survivor care delivery models are needed to improve care processes and health outcomes. This pilot trial will test the ACT program, which uses a multidisciplinary model focused on primary care to address this gap.

**Trial Registration:**

ClinicalTrials.gov NCT05184894; https://www.clinicaltrials.gov/ct2/show/NCT05184894

**International Registered Report Identifier (IRRID):**

DERR1-10.2196/48109

## Introduction

Acute kidney injury (AKI) affects nearly 20% of hospitalized patients. It is associated with a 6.5-fold higher mortality, a 3.5-day longer length of stay, and US $5 billion in annual hospital costs in the United States. Chronic kidney disease (CKD) develops in 15%-30% of AKI survivors and 49% are readmitted to the hospital within 1 year. These complications decrease patients’ quality of life and strain health care resources. This significant risk of poor outcomes can be partially attributed to gaps in kidney-focused care and education during transitions. One study found that 30% of AKI survivors failed to receive basic laboratory and clinical follow-up of kidney health after discharge and that avoidable nephrotoxin exposure during this period independently increased the risk for chronic kidney disease [1,2]. Another study showed that laboratory monitoring of serum creatinine and urine protein, which is recommended as best practice, was only done in 54% and 14%, respectively, of AKI survivors at 6 months [3]. These care gaps place patients at risk for exposure to potentially modifiable determinants of long-term complications, such as nephrotoxins. A large population-based cohort study showed that 74% of AKI survivors received 1 or more nephrotoxins at hospital discharge. This was associated with a 1.4-fold increase in risk for new or worsening CKD and compounded 1.13-fold with each additional nephrotoxin [2].

Survivorship programs facilitating the transition from inpatient to outpatient care have improved clinical and patient-centered outcomes in patients with complex care needs, such as cancer and critical illness survivors [4-7]. Though experience with AKI survivor care transition programs is limited, the most frequently proposed model is a dedicated nephrologist-led clinic to deliver core components of best practices for post-AKI care. These have been associated with improved patient kidney health knowledge and process outcomes, such as timely serum creatinine monitoring after discharge [8,9]. Despite promising results in initial reports, concerns exist about scalability, acceptability of patients, and generalizability to rural and low-income populations where specialty nephrology care is often unavailable [10,11]. Patients are typically seen more than 1 month after discharge, increasing the risk for nephrotoxin exposure and loss of follow-up. Additionally, specialty consultation may not be needed for all patients and may introduce additional fragmentation of care, costs, and treatment burden. Nephrologists have called for the integration of other disciplines to enhance AKI survivor care delivery capacity [12], including primary care providers (PCP) to evaluate laboratory tests and perform a kidney function assessment early in the postdischarge period [13].

To address these concerns, we developed the multidisciplinary AKI in Care Transitions (ACT) program, which embeds post-AKI care in the patient’s medical home, primary care. The bundled intervention includes (1) the use of an electronic health record (EHR) alert to identify high-risk AKI survivors, (2) education and care coordination from nurses in nephrology before discharge, and (3) a posthospital visit with a PCP and a pharmacist within 14 days after discharge. Nephrology referral is coordinated based on the needs and preferences of the patient, inpatient care providers, and the patient’s PCP. Pilot testing revealed higher 14- and 30-day cumulative incidence of laboratory and provider follow-up in ACT program participants compared to those receiving usual care. In addition, participants who received kidney health education improved their understanding of AKI and its consequences.

This pilot trial aims to test the feasibility of study procedures for a future hybrid effectiveness and implementation trial, including patients’ willingness to be enrolled and randomized, participant retention, and chosen outcome measures. We will also assess the feasibility and acceptability of participation in the ACT program and identify determinants of successful implementation into routine clinical care.

## Methods

### Overview

**Figure 1:**
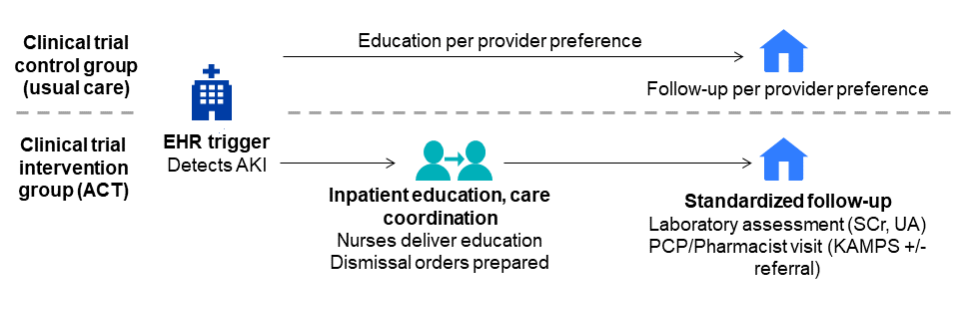
Overall study design. ACT: acute kidney injury in care transitions; AKI: acute kidney injury; EHR: electronic health record; KAMPS: kidney health follow-up framework (Textbox 1); PCP: primary care providers; SCr: serum creatinine; UA: urinalysis.

In preparation for a future large-scale clinical trial to test the effectiveness and implementation of the ACT program for improving care quality and outcomes, this study will evaluate the feasibility and acceptability for patients and staff (aim 1) and gather preliminary effectiveness data (aim 2). To accomplish these aims, we will conduct a pilot randomized trial using a convergent mixed methods approach (Figure 1). We will use the Consolidated Framework for Implementation Research (CFIR) [14] to systematically assess potential barriers and facilitators (ie, determinants) that influence implementation outcomes.

### Ethics Approval

The study was reviewed and approved by the Mayo Clinic Institutional Review Board (21-011055) on December 14, 2021, and was registered at ClinicalTrials.gov (NCT05184894). This study will employ a Data Safety and Monitoring Plan to monitor participant safety, data completeness and adherence to study protocol, data integrity, participant privacy, data confidentiality, and study documentation.

### Setting and Participants

This pilot trial was conducted at Mayo Clinic, an academic medical center in Rochester, Minnesota. Mayo Clinic is an integrated health care delivery system delivering primary and specialty care to local, regional, national, and international patients. Mayo Clinic primary care practices provide comprehensive primary care to approximately 150,000 empaneled patients —local area residents, Mayo Clinic employees and their dependents—cared for at 7 full-service clinical sites and 2 express care sites in Rochester and surrounding counties. It employs a team-based care model that includes physicians, advanced practice providers (nurse practitioners and physician assistants), nurses, and embedded clinical pharmacists who consult with patients independently or in collaboration with the PCP. At Mayo Clinic, no formal dedicated AKI-survivor clinic exists.

Mayo Clinic Rochester Hospital has 5 inpatient nephrology consultation services that include nurse educators whose primary role is educating hospitalized patients discharged on dialysis. Patients can be referred for nephrologist follow-up in a Rochester clinic by inpatient care teams, including the nephrology consult services, or any other inpatient or outpatient provider.

Patient participants for the ACT trial are identified using an EHR screening alert, a validated electronic AKI surveillance tool that uses serum creatinine and urine output to detect AKI [15]. Eligible individuals have stage 3 AKI (severe) based on consensus criteria [16] during their hospitalization, a PCP at Mayo Clinic in Rochester, do not need dialysis at discharge, and are not discharged to hospice. Patients who are unable to participate (eg, clinician-documented dementia in the EHR or with limited English proficiency) or who do not provide informed consent are excluded. Recipients of any transplant or acute cellular therapy within 100 days of enrollment are also excluded. Eligibility is determined using the EHR screening alert and through manual chart review by a study team member. Individuals may only be enrolled in the trial one time. Remuneration is not provided to any participant.

Patient participants in the intervention group and staff involved in delivering components of the ACT program are invited to participate in semistructured interviews or observations of ACT program interactions (eg, patient education, provider visit) to explore the feasibility and acceptability of the ACT program. Participants are sampled on key factors (eg, age, urban or rural geographic location, and ACT program role) to facilitate the selection of information-rich cases while capturing major variations based on patient factors or staff background or experience.

Patients provide written informed consent for trial enrollment and indicate whether they consent to be contacted for an interview and/or have their visit observed. Oral consent is obtained from clinicians before participating in interviews or observations. Clinicians are approached for oral consent at the first study encounter they are involved in. This consent carries forward for all subsequent encounters (eg, with other patients). Observations are performed using video and audio recordings. At any time once enrolled, including during the visit, a patient or clinician may withdraw their consent for participation in the trial, observations, or an interview. Declining participation in 1 aspect of the trial does not preclude ongoing participation in others.

### Study Groups

#### Participant Identification

A study team member contacts potential patient participants before discharge to obtain written informed consent for the trial. Patients are approached approximately 48-72 hours before the estimated discharge date. Participants are randomized 1:1 to either the control or intervention group. Individuals are randomly allocated equally to the intervention or control groups using a permuted block design with varying block sizes between 2 and 4. The study statistician set up the randomization scheme and provided numbered envelopes to the study coordinator with the randomly assigned group enclosed. After completing the informed consent, the study coordinator opens the envelope and discloses the group allocation to participants.

#### Intervention Group

Participants in the intervention group receive a care bundle inclusive of education, care coordination, and postdischarge follow-up. Before discharge, participants receive a consultation from nephrology nurse educators. These nurses provide kidney health education, as previously described [17], which is tailored to the patient’s needs. To ensure consistency, nurses aim to achieve understanding using teach-back questions. Scheduling coordinators place orders for postdischarge laboratory tests (serum creatinine and urine study for protein assessment) and outpatient visits with a PCP and a pharmacist for signature by the primary inpatient service before discharge. Laboratory and provider visits are ideally scheduled within 14 days of discharge [4]. PCP and pharmacist encounters can occur in person or through video (PCP or pharmacist visits) or telephone (pharmacist visits only). Structured around the KAMPS framework (Textbox 1 [18, 19]), the PCP and pharmacist provide patient education, medication reconciliation, and disease state management with specialist referral as needed.

**KAMPS framework for components of kidney follow-up care [18,19].**

**Framework**

- K: Kidney function assessment with laboratory testing
- A: Awareness and education
- M: Medication reconciliation and review
- P: Individualized blood pressure monitoring
- S: Sick day education

It is expected as part of standard care that the PCP and pharmacist review the range of care needs for the patient in the transitional period, including, but not limited to AKI. Pharmacists discuss recommendations with the provider in person, if possible, or through secure message. Clinical decision support tools have been developed and embedded in the EHR [17], including descriptions of the KAMPS framework domains and links to additional kidney care resources through a proprietary medical knowledge system [20]. Orders for outpatient nephrology follow-up, if indicated, may be placed by the inpatient care teams or the PCP. Participants in this group are also invited to participate in semistructured interviews.

#### Control Group

Participants receive no specific study-related intervention. AKI identification, patient education, and follow-up care are at the discretion of the primary treating team. It is customary for these patients to receive some degree of laboratory monitoring and clinical follow-up in the postdischarge period, but for this group, timing and components are not standardized. Care providers have access to the same educational materials for providers and patients as in the intervention group.

### Data Collection

#### Overview

Individuals that consent to participate will be captured in the REDCap system. Those found to be ineligible or eligible but declining participation will be captured in a recruitment tracking log. If spontaneously offered, reasons for declining participation will be recorded, but these will not be deliberately probed due to regulatory limitations. The Mayo Clinic Unified Data Platform will be used to abstract electronically available data. The Unified Data Platform is an aggregated data resource inclusive of current and legacy EHR and administrative billing data across the Mayo Clinic enterprise. Other sources of data include interviews and direct observations of clinical encounters. An overview of the data collection plan is outlined in Table 1.

**Table 1:** Summary of study measures and assessment timing.

Element	Source	Hospital admission	Randomized	Hospital discharge	Study follow-up visit (ACT^a^ patients only)	30 days	90 days	1 year
Sociodemographic factors, comorbidities	UDP^b^	✓						
Attributes of hospitalization and AKI^c^ episode	UDP			✓				
Medication use	UDP	✓		✓		✓		
Brief health literacy screen	Patient self-report		✓					
mKiKS^d^	Patient self-report		✓			✓		
PROMIS 10	Patient self-report		✓			✓		
Was it worth it?	Patient self-report					✓^e^		
Encounter observation	Audio or videorecording				✓^e^			
Qualitative interviews	Patient or staff self-report					✓^e,f^		
MAKE^g^, unplanned ED^h^ visit, rehospitalization	UDP						✓	
CKD^i^ or death	UDP							✓
Kidney laboratory assessments, outpatient visits	UDP						✓	

^a^ACT: AKI in Care Transitions.

^b^UDP: Unified Data Platform, source for quantitative data collection unless otherwise specified.

^c^AKI: acute kidney injury.

^d^mKiKS: modified Kidney Knowledge Survey.

^e^Intervention patients or clinicians only.

^f^Staff participating in interviews will complete the Feasibility of Intervention Measure and Acceptability of Intervention Measure surveys [21].

^g^MAKE: major adverse kidney event.

^h^ED: emergency department.

^i^CKD: chronic kidney disease.

#### Quantitative Data

Data will be abstracted from the Mayo Clinic UDP on patient demographics, comorbidities, attributes of the hospitalization, details about the episode of AKI and the degree of kidney function recovery by discharge, follow-up processes of care (laboratory assessments and visits), and clinical outcomes. Health literacy is evaluated in all participants at baseline using the Brief Health Literacy Screen (Multimedia Appendix 1) [22]. Select medication data will be collected at baseline (preadmission based on the documented medication list in the EHR), discharge, and 30-day follow-up. This will include the use of angiotensin-converting enzyme inhibitors and angiotensin receptor blockers, sodium-glucose cotransporter 2 inhibitors, metformin, glucagon-like peptide 1 receptor antagonists, sulfonylureas, insulin, loop diuretics, thiazide diuretics, statins, aspirin, and nonsteroidal anti-inflammatory drugs. The total number of medications at discharge will be evaluated. Using previously described methods, we will review pharmacist documentation of medication discrepancies and drug therapy problems for any patient completing a pharmacist visit [4]. Drug therapy problems will be categorized as pertaining to effectiveness, indication, safety, or adherence and relationship to renoprotective or nephrotoxic medications (Textbox 2). Drug therapy problems will be assessed and categorized by a single pharmacist with 11 years of experience and advanced credentialing as a medication management services pharmacist specializing in primary care.

**Medications classified as nephrotoxic or renoprotective. Medication data will be collected at baseline (ie, preadmission medications), hospital discharge, and 30-day outpatient follow-up.**

**Nephrotoxic medications**

- Antivirals: Acyclovir, ganciclovir, tenofovir, valacyclovir, foscarnet
- Antibiotics: Aminoglycosides, ciprofloxacin, colistimethate, nafcillin, oxacillin, penicillin, polymyxin, rifampin, trimethoprim/sulfamethoxazole, vancomycin (intravenous product only), piperacillin/tazobactam, ticarcillin or clavulanate
- Immunosuppressants: tacrolimus, sirolimus, cyclosporine
- Chemotherapeutics: Carboplatin, cisplatin, cyclophosphamide, ifosfamide, imatinib, irinotecan, gemcitabine, ibrutinib, oxaliplatin, lenalidomide, melphalan, sorafenib, temozolomide, topotecan, methotrexate (>20 mg/week)
- Other: Amphotericin B, lithium, mesalamine, pamidronate, sulfasalazine, topiramate, zonisamide, zoledronic acid, proton pump inhibitors, nonsteroidal anti-inflammatory drugs (includes aspirin doses >325 mg/day)

**Renoprotective medications**

- Angiotensin-converting enzyme inhibitors
- Angiotensin receptor blockers
- Sodium-glucose cotransporter 2 inhibitors
- GLP-1 receptor agonists

#### Qualitative Data

Interviews will be conducted by a female study team member with expertise in qualitative methods (DMF) who has no prior relationship with participants. Interviews begin with a brief description of the ACT program, the study’s goals, and information about informed consent, which is documented. CFIR domains informed the creation of 1 semistructured interview guide for patients and 1 for staff (Multimedia Appendix 1), where questions are tailored to each role (eg, PCP, pharmacist, and scheduling coordinator). Interviewees will be asked to describe their experiences with the ACT program and their sense of the outcomes. Interviews will be conducted over the phone and recorded and transcribed with permission. Transcripts will be reviewed by 2 additional study team members and content experts (HPM and EFB), with the refinement of the interview guide as needed.

Two study team members, including 1 with training in qualitative methods (HPM), will review observations to assess the impact of the ACT interventions on the clinical encounter. Reviewers will watch the video (when available) or read the transcripts of the encounter. Using an observation template for field notes, encounters will be primarily evaluated for the 5 elements of KAMPS framework (Textbox 1) [19,23]. The duration spent on each KAMPS framework element and discussion about kidney health (relative to other conditions) will be cataloged. Reviewers will record whether they observed the clinician using the ACT clinical decision support tools or any print or electronic materials about AKI or kidney health. Occasions where kidney health is discussed will be cataloged as clinician-initiated or patient-initiated.

Study team members will use freeform field notes throughout the study to document notable observations during interactions with patient and staff participants (eg, participating patients failing to recall study enrollment or their episode of AKI).

### Outcomes

#### Feasibility, Fidelity, and Acceptability

To assess the feasibility of patients’ willingness to be enrolled and randomized, we will evaluate the number of patients screened, approached for consent, and randomized during the study time frame (intention-to-treat group). Intervention fidelity will be measured by the proportion of participants in the intervention group who complete the nurse education, laboratory testing, pharmacist visit, and PCP visit (per-protocol group). For those who do not complete the full ACT intervention, we will characterize the attrition (eg, inability to deliver education during hospitalization, inability to schedule postdismissal visits, and patient did not complete the postdismissal visits), which reflects the feasibility of incorporating the ACT program into routine transitional care. We will also evaluate the proportion of participants in the ACT group where clinicians interfaced with clinical decision support alerts using electronic audit trails. Staff participating in interviews will complete the Feasibility of Intervention Measure and Acceptability of Intervention Measure surveys, validated tools to measure implementation outcomes [21]. Acceptability of the ACT program will also be determined through patient completion of the “Was it worth it?” survey at follow-up (Multimedia Appendix 1) [24], qualitative interviews with consenting staff and patients, and observations of the clinical encounter.

#### Patient Knowledge and Quality of Life

We will evaluate patient knowledge about AKI measured with an adaptation of the Kidney Knowledge Survey (modified KiKS; mKiKS). mKIKS assesses participants’ objective knowledge about AKI causes, risk factors, and management [25]. Unanswered questions (missing data) on the mKiKS are assigned a score of 0, and the total score is used for comparisons (Multimedia Appendix 1). Quality of life will be assessed using the PROMIS Global 10 tool v1.2 (Multimedia Appendix 1). Patient-reported outcomes will be collected in person or over the phone by a study team member.

#### Process and Clinical Outcomes

Participants will be followed for 1 year post discharge or until death or loss of follow-up within that time frame. Process outcomes include kidney laboratory assessments and their timing, clinician visits (eg, with the PCP, pharmacist, and nephrologist), visit type, and timing (in-person vs telemedicine or web-based). At 90 days, clinical outcomes for assessment include major adverse kidney event, emergency department visits, or unplanned rehospitalization. Incidence of de novo or progressive chronic kidney disease or death up to 12 months after discharge will be documented. The number of identified nephrotoxic or renoprotective medications, medication discrepancies, and drug therapy problems will be described.

### Data Analysis

#### Quantitative Data

To describe the feasibility of ACT and generate preliminary estimates of the effect on patient-reported and clinical outcomes, descriptive analyses will be conducted in three groups: (1) the intention-to-treat population (anyone randomized), (2) the per-protocol population (randomized patients who receive all components of the study intervention), and (3) the complete follow-up population (the per-protocol population with follow-up patient-reported outcomes at 30±7 days). Baseline characteristics will be described with means (SDs) and counts and percentages. Comparisons will use the *t* test for continuous data and the chi-square test for categorical data. Any baseline imbalances (*P*<.05) will be explored as a possible factor to adjust for when the outcome measures are analyzed. Clinical outcomes up to 12 months will be compared with Cox proportional hazards models.

AKI knowledge will be described between groups with the mean mKiKS score. Model imputation will be used for patients with incomplete follow-up. Mean between-group differences in the total baseline and follow-up scores will be compared using the *t* test or nonparametric Mann-Whitney *U* test. An exploratory multivariable linear regression model will be fit with group assignment as the independent predictor of interest, mean follow-up knowledge scores as the dependent variable, and baseline scores as a covariate. Other variables to be considered will include age, sex, baseline health literacy, and baseline comorbidities, specifically CKD.

#### Qualitative Data

Interview transcripts will be uploaded to NVivo, software that assists in qualitative data organization and analysis. Five interview transcripts will be reviewed by 2 qualitative methods experts (DMF and JMG) and 2 content experts (HPM and EFB) to develop an initial codebook, which will be iteratively refined as it is applied to all interview data by 2 independent coders (DMF and HPM). Codes will be deductive (informed by CFIR domains) and inductive (identification of emerging concepts) and used in a framework analysis conducted by experts in qualitative methods (DMF and JMG) and post-AKI care (HPM and EFB). Themes will be developed through discussion and consensus and compared to ensure they capture the full range and depth of interview data. Themes will be compared between data types (eg, staff roles) and assessed for areas of complementarity (eg, domains describing interrelated factors), concordance, and discordance.

Twenty percent of the recorded encounters will be reviewed independently and in duplicate by 2 study team members. Concordance between individuals in assigned ratings of the 5 elements of the KAMPS framework will be evaluated. If greater than 0.8, reviewers will be considered concordant, and 1 study team member will evaluate the remaining encounters. If concordance is not met, an additional 20% of the included encounters will be evaluated in duplicate.

#### Data Integration

Quantitative data on feasibility and acceptability will be supported by qualitative data and integrated using an embedding approach. Herein, findings from qualitative interviews will supplement results by providing rich data on patient and staff experiences and perspectives on participating in the ACT program. Data will be integrated at the conclusion of the trial. Divergence (ie, inconsistent or conflicting findings) will be examined through refined data analysis and reconciliation, where results are reviewed with a deliberate focus on understanding inconsistencies. Results will be reported using a weaving narrative and joint display tables.

#### Sample Size

Pilot data estimates that approximately 1-2 patients per week will be identified with the EHR trigger, of which 70% will be eligible, agree to participate in the trial, and have evaluable outcomes data. We will assess the feasibility of study recruitment and randomization and the chosen outcome measures through comparison with these estimates. We project that a sample size of 25 patients per group is needed to assess the feasibility and acceptability of participating in the ACT intervention. Thus, we aim to enroll 50 total patients during an 18-month study time frame.

## Results

This study received funding from the Agency for Healthcare Research and Quality on April 21, 2021. Institutional Review Board approval and study commencement were delayed due to unavoidable challenges associated with the COVID-19 pandemic. Following Institutional Review Board approval on December 14, 2021, the first participant was enrolled on January 24, 2022. As of March 14, 2023, seventeen participants each have been enrolled in the intervention and usual care group (total 34).

## Discussion

### Overview

Nephrologists have called for integrating other disciplines to enhance AKI survivor care delivery capacity [12]. Nephrologists and PCPs support early laboratory monitoring and kidney function assessment in primary care, and PCP follow-up is considered a cornerstone of care continuity [13]. PCPs have a preexisting rapport with patients and caregivers, familiarity with all the patient’s medical conditions, and insights into both clinical and nonclinical factors affecting a patient’s health and health care. They may also provide less expensive and more accessible care for the health system [5]. As part of comprehensive primary care, the contribution of other allied health team members to care delivery is a recognized facilitator of high-quality, patient-centered care [19,26]. Nurses delivering kidney health education and coordinating post-AKI care have improved patient participation [9] and kidney health knowledge [27]. Likewise, pharmacists are uniquely qualified to complete a detailed medication reconciliation and identify medication therapy problems during the dynamic arc of kidney function recovery [28]. Pharmacist-PCP collaboration can reduce hospital readmission and the potential for medication harm compared to care with a PCP alone [4,29]. Despite widespread support for a multidisciplinary approach to post-AKI care, there is a paucity of data describing the incorporation of nonnephrologists into health care delivery models. The ACT program is poised to address common barriers faced by AKI survivors transitioning from the hospital to home through a patient-centered approach integrated in primary care.

Limitations of the proposed study must be acknowledged. The primary outcome of the trial is feasibility and acceptability, thus the sample size is modest. The data will be collected to gather preliminary estimates on clinical outcomes to inform a future trial, but this study is not expected to provide definitive insight into clinical outcomes. All participants will receive primary care in the same region as the tertiary care center where they are hospitalized for AKI, and all sites use a shared EHR. Although this minimizes the likelihood of missing follow-up data, it is unknown how these findings may translate to patients receiving primary care at a greater distance or in practices that do not share an EHR with the discharging hospital.

### Conclusion

The ACT program uses a multidisciplinary, coordinated care transition model to enhance kidney health, knowledge, and safety in high-risk patients with existing gaps in care quality. Results from this trial will indicate the feasibility of recruitment and retention, study procedures, chosen outcomes, and the acceptability of the ACT program to patients and staff participants. In addition, this will determine the suitability of the ACT program for further large-scale testing, with the ultimate goal of improving patient and provider experience and outcomes.

## Appendix

Multimedia Appendix 1 Instruments and guides.

## Abbreviations

ACT: Acute Kidney Injury in Care Transitions
AKI: acute kidney injury
CFIR: Consolidated Framework for Implementation Research
CKD: chronic kidney disease
EHR: electronic health record
mKiKS: modified Kidney Knowledge Survey
PCP: primary care provider

## Data Availability

Source data may be made available upon reasonable request.
