# Supplementary material for: Comprehensive Acute Kidney Injury Survivor Care: Protocol for the Randomized Acute Kidney Injury in Care Transitions Pilot Trial
Source: JMIR Res Protoc. 2023 May 22;12:e48109. doi: 10.2196/48109 (PMC10242466; doi:10.2196/48109)
Supplement: Multimedia Appendix 1 [file resprot_v12i1e48109_app1.pdf]

Supplementary Appendix

Brief Health Literacy Screen

Modified Kidney Knowledge Survey (mKiKS)

PROMIS Global Health v1.2

Was It Worth It

Provider Interview Guide

Patient Interview Guide

Brief Health Literacy Screen

1. **How often do you have someone help you read hospital materials? (select one)**
    a. Always
    b. Often
    c. Sometimes
    d. Occasionally
    e. Never

2. **How confident are you filling out medical forms by yourself? (select one)**
    f. Not at all
    g. A little bit
    h. Somewhat
    i. Quite a bit
    j. Extremely

3. **How often do you have problems learning about your medical condition because of difficulty understanding written information? (select one)**
    k. Always
    l. Often
    m. Sometimes
    n. Occasionally
    o. Never

4. **How often do you have a problem understanding what is told to you about your medical condition? (select one)**
    p. Always
    q. Often
    r. Sometimes
    s. Occasionally
    t. Never

Total Score: ______

Each question is scored on a 5-point response scale and is summed to produce a total score ranging from 4 to 20. This score is divided into inadequate (4–12), marginal (13–16), and adequate (17–20) categories.

Modified KiKS (mKiKS)

1. **Did you experience acute kidney injury, or damage to your kidney function, while in the hospital? (select one)**
    a. No
    b. Yes – I had mild AKI
    c. Yes – I had severe AKI
    d. Unsure

2. **AKI is when (select one)**
    a. Your blood counts drop too low
    b. Your kidneys suddenly stop working well
    c. Your back hurts suddenly
    d. Your blood pressure is too high

3. **Common causes of AKI include (select all that apply)**
    a. Taking ibuprofen
    b. Being dehydrated
    c. Eating too much salt
    d. Kidney stones
    e. Getting a severe infection
    f. Taking acetaminophen

4. **AKI is identified by measuring (select one)**
    a. Creatinine levels in your blood
    b. Your blood pressure
    c. An X-ray
    d. Your cholesterol

5. **What might be an early sign of AKI (select one)**
    a. Fever
    b. Not making much urine
    c. Back pain
    d. Headache

6. **Having AKI puts you at risk for (select one)**
    a. Liver problems
    b. Weight loss
    c. Readmission to the hospital
    d. Headaches

7. **What can you do to help reverse the effects of AKI and protect your kidney health (select all that apply)**
    a. Limit your alcohol intake
    b. Stop smoking
    c. Eat more red meat
    d. Keep appointments with your healthcare team
    e. Maintain a healthy weight
    f. Control your blood pressure
    g. Control your blood sugar
    h. Avoid dairy products
    i. Discuss with your doctor or pharmacist before taking new medications
    j. Avoid over-the-counter medications like ibuprofen or naproxen

Total Score: ______

Each correct answer on questions 2-7 is assigned 1 point. In questions with multiple correct answers, 1 point will be awarded for each correct answer identified. There will be no negative points for incorrect choices. Scores will be calculated by dividing the number correct answer choices by the total possible (n=16).

PROMIS Global Health v1.2

1. **In general, would you say your health is…**
    a. Excellent
    b. Very good
    c. Good
    d. Fair
    e. Poor

2. **In general, would you say your quality of life is…**
    a. Excellent
    b. Very good
    c. Good
    d. Fair
    e. Poor

3. **In general, how would you rate your physical health?**
    a. Excellent
    b. Very good
    c. Good
    d. Fair
    e. Poor

4. **In general, how would you rate your mental health, including your mood and your ability to think?**
    a. Excellent
    b. Very good
    c. Good
    d. Fair
    e. Poor

5. **In general, how would you rate your satisfaction with your social activities and relationships?**
    a. Excellent
    b. Very good
    c. Good
    d. Fair
    e. Poor

6. **In general, please rate how well you carry out your usual social activities and roles. (This includes activities at home, at work and in your community, and responsibilities as a parent, child, spouse, employee, friend, etc.) …**

a. Excellent
b. Very good
c. Good
d. Fair
e. Poor

7. **To what extent are you able to carry out your everyday physical activities such as walking, climbing stairs, carrying groceries, or moving a chair…**
    a. Completely
    b. Mostly
    c. Moderately
    d. A little
    e. Not at all

8. **In the past 7 days, how often have you been bothered by emotional problems such as feeling anxious, depressed, or irritable?**
    a. Never
    b. Rarely
    c. Sometimes
    d. Often
    e. Always

9. **How would you rate your fatigue on average**
    a. None
    b. Mild
    c. Moderate
    d. Severe
    e. Very severe

10. **How would you rate your pain on average from 0 being no-pain to 10 being the worst pain imaginable?** ______________________

Raw Score:_______
T-Score: _______
Physical Health Score: _______
Mental Health Score:_______

Raw scores are summed and then converted into standardized T-scores using provided tables. The distributions of T-scores are such that 50 represents the average (mean) for the US general population with a standard deviation of 10. Physical health scores are derived from questions 3, 7, 9, 10 (items 3, 6, 7, and 8 in PROMIS PDF). Mental health scores are derived from questions 2, 4, 5, and 8 (items 2, 4, 5, and 10 in PROMIS PDF).

Was It Worth It

1. **The overall ACT intervention was worthwhile**
    a. Strongly disagree
    b. Disagree
    c. Neutral
    d. Agree
    e. Strongly agree

2. **The nurse education I received in the hospital was worthwhile**
    a. Strongly disagree
    b. Disagree
    c. Neutral
    d. Agree
    e. Strongly agree
    f. Unable to assess/Not applicable

3. **The primary care provider/pharmacist visit after I left the hospital was worthwhile**
    a. Strongly disagree
    b. Disagree
    c. Neutral
    d. Agree
    e. Strongly agree
    f. Unable to assess/Not applicable

4. **The ACT intervention made it easier for me to participate in my kidney care**
    a. Strongly disagree
    b. Disagree
    c. Neutral
    d. Agree
    e. Strongly agree
    f. Unable to assess/Not applicable

5. **I would recommend the ACT intervention to others**
    a. Strongly disagree
    b. Disagree
    c. Neutral
    d. Agree
    e. Strongly agree
    f. Unable to assess/Not applicable

6. **My quality of life got better by receiving the ACT intervention**
   a. Strongly disagree
   b. Disagree
   c. Neutral
   d. Agree
   e. Strongly agree
   f. Unable to assess/Not applicable

<u>Provider Interview Guide</u>

Context

To start, could you tell me a little bit about yourself and your background?

- How long have you been in practice?
- What is your role on the care team?
- What types of patients do you usually interact with?

Barriers and Facilitators

I'd like to start by asking about specific aspects of the ACT program that you may have participated in.

- **Nurses**: Part of the ACT program involves <u>delivering patients and caregivers education</u> that is specific to AKI before they leave the hospital. Do you recall a situation where you provided this type of education? If yes, what was the process of delivering education like?
    - What did you think of the timing of education during hospitalization?
    - What was your interaction with patients/caregivers like?
    - What did you think of the materials that were available and that you used?

- **Scheduling Coodrinator**: The ACT program involves <u>preparation of dismissal orders for labs and a post-hospital follow-up visit with a primary care provider and pharmacist</u>. Do you recall a situation where you were asked to schedule these orders by a member of the ACT team, likely Erin Barreto or Angie Tinaglia? If yes, what was the process of preparing and coordinating these orders like?
    - What did you think of the timing of order placement during hospitalization?
    - What was it like placing the orders and getting them signed?
    - How was your experience scheduling the follow-up?

- **Physicians/APPs**: Patients in the ACT program undergo urine studies, serum creatinine and electrolyte monitoring, and have a visit with a pharmacist, usually before their scheduled visit with you in primary care. There is also a Best Practice Advisory in their Epic record available for you to view that suggests activities related to post-AKI care, such as kidney function assessment and individualized blood pressure monitoring. Do you recall seeing any patient who meets any of these criteria? If yes, what was it like delivering their care?
    - What was the interaction with the pharmacist like?
    - What did you think of the interaction with best-practice advisories?
    - What did you think of the ways laboratory assessments were coordinated? And what was your experience like with their interpretation?
    - How did you feel about the way post-AKI care was attended to alongside other patient conditions/needs during the visit?
    - What effect did the patient's enrollment in the ACT program have on your documentation or their scheduling?

- **Pharmacists**: Patients in the ACT program undergo urine studies, serum creatinine and electrolyte monitoring, and visits scheduled with a pharmacist and primary care provider after discharge. There is also a Best Practice Advisory in their Epic record available for you to view that suggests activities related to post-AKI care, such as kidney health education, medication review for nephrotoxins, individualized blood pressure monitoring, and dot phrase about post-AKI care that assists in progress note documentation. Do you recall seeing a patient who meets any of these criteria? If yes, what was it like delivering their care?
    - What was the interaction with the provider like?
    - What did you think of the interaction with best-practice advisories?
    - What did you think of the ways laboratory assessments were coordinated? And what was your experience like with their interpretation?
    - How did you feel about the way post-AKI care was attended to alongside other patient conditions/needs during the visit?
    - What effect did the patient's enrollment in the ACT program have on your documentation or their scheduling?

What aspects of the ACT program went well?

What challenges did you encounter with the ACT Program?

- What contributed to these, in your opinion? *(Probe to try to get at the 'why' if not already specified, focusing on implementation barriers and potential solutions)*
- What did you think of the communication about patients enrolling in or participating in the program?
- What strategies do you think would help address the challenges you identified?
- **For time constraints concerns**: We know that time constraints are something that busy clinicians, like yourself, face frequently. What changes to the ACT program would you suggest to address that hurdle?

To what extent do you feel the ACT program addresses the needs of AKI survivors?

What other suggestions do you have for how ACT be improved to better care for AKI survivors?

What would help you feel more prepared to participate effectively in the ACT workflow?

Feasibility of Assessment Measure (all providers)

	Completely disagree	Disagree	Neither agree nor disagree	Agree	Completely agree
1. The ACT program seems implementable.	✍	✍	✍	✍	✍
2. The ACT program seems possible.	✍	✍	✍	✍	✍
3. The ACT program seems doable.	✍	✍	✍	✍	✍
4. The ACT program seems easy to use.	✍	✍	✍	✍	✍

Acceptability of Assessment Measure (all providers)

	Completely disagree	Disagree	Neither agree nor disagree	Agree	Completely agree
1. The ACT program meets my approval.	✍	✍	✍	✍	✍
2. The ACT program is appealing to me.	✍	✍	✍	✍	✍
3. I like The ACT program.	✍	✍	✍	✍	✍
4. I welcome The ACT program.	✍	✍	✍	✍	✍

Patient Interview Guide

Context

To start, could you tell me a little bit about yourself and your background?

- How would you characterize your experience with healthcare recently?

Perspectives, experiences

Thinking back to when you were in the hospital and right after you got out of the hospital. Can you give me your thoughts on how kidney health and kidney function were discussed in the hospital and shortly after hospitalization by your care team?

Barriers and Facilitators

People in the ACT program get education on kidney health before leaving the hospital. After being discharged from the hospital, they have urine and blood tests and scheduled clinic visits with a primary care provider and pharmacist to monitor the kidneys.

What aspects of the ACT program do you remember participating in, if any? What was your experience with that?

Don't worry if you can't recall them just now, we can ask you about each.

- Usually, people receive education on several topics during their hospitalization. Do you recall receiving education prior to discharge about your kidney health? If yes, what was your experience with that?
- In the one month or so after you left the hospital, did you have blood or urine tests performed? Do you know if any of them were to monitor your kidneys?
    - If No: Can you remember if these were scheduled for you? If they were scheduled but you did not attend the appointment, can you share what made participating in that challenging?
    - If Yes: What was your experience with that?
    - **If challenges identified**: What strategies do you think would help address those challenges?
- Were you able to attend a clinic visit with a primary care provider about your kidney health after you got out of the hospital?
    - If No: Can you share what made participating in that challenging?
    - If Yes: What was your experience with that?
    - **If challenges identified**: What strategies do you think would help address those challenges?
- Do you recall meeting with a pharmacist as part of your follow-up care?
    - If Yes: What was your experience with that?
    - **If challenges identified**: What strategies do you think would help address those challenges?
- Have you and your primary care provider ever talked about the need to see a kidney specialist?
    - If yes: What things came up during that discussion?
    - **If challenges identified**: What strategies do you think would help address those challenges?

Tell me what you remember about the plan for your kidney care after you were hospitalized

- Tell me about any instructions you might have received about your medications?
- Tell me about any information you received about changes in diet or lifestyle habits that were related to kidney health?

Being in the hospital can be overwhelming, especially when you are receiving a new diagnosis or new information. What would have helped you to better understand what was going to happen about your kidney care after you left the hospital?

How do you feel ACT program's in-hospital education and outpatient kidney health monitoring, with labs and clinic visits, met your kidney care needs?

To what extent was a family member or caregiver involved in activities related to the ACT program, such as in-hospital education or post-dismissal visits?

- What could be done to more effectively engage them or meet their needs?

If someone you know had an acute kidney injury while in the hospital, how could the ACT program be improved to better care for them? If they asked you what part of the ACT program was most helpful, what would you share with them?
